# Vasculogenic mimicry-associated ultrastructural findings in human and canine inflammatory breast cancer cell lines

**DOI:** 10.1186/s12885-019-5955-z

**Published:** 2019-07-30

**Authors:** Lucía Barreno, Sara Cáceres, Ángela Alonso-Diez, Ana Vicente-Montaña, María Luisa García, Mónica Clemente, Juan Carlos Illera, Laura Peña

**Affiliations:** 1 Veterinary Clinical Hospital, Pathology Service, Complutense University of Madrid, Madrid, Spain; 2 Department of animal Physiology, Complutense University of Madrid, Madrid, Spain; 3 National Center of Electron Microscopy, Complutense University of Madrid, Madrid, Spain

**Keywords:** Vasculogenic mimicry, Inflammatory breast cancer, Mammospheres, Canine, Electron microscopy, Comparative oncology

## Abstract

**Background:**

Human inflammatory breast cancer (IBC) and canine inflammatory mammary cancer (IMC) are the most lethal mammary cancers. An exacerbated angiogenesis and the existence of vasculogenic mimicry (VM) are hallmarks of these tumors. The information regarding VM and ultrastructural characteristics of mammary cell lines is scant.

**Methods:**

In this study, IBC cell line SUM149 and IMC cell line IPC-366 in adherent (2D) and non-adherent (3D) (mammospheres, cancer stem cells) conditions were analyzed by transmission and scanning electron microscopy (TEM and SEM, respectively).

**Results:**

The TEM revealed round to oval shape cells with microvilli on the surface, high numbers of peroxisomes in close apposition to lipid droplets and some extracellular derived vesicles. The TEM and the SEM mammospheres revealed group of cells clumping together with a central lumen (resembling a mammary acini). The cells joint are tight junctions and zonula adherens. By SEM two cell morphologies were observed: spherical and flattened cells. There was evidence endothelial-like cells (ELCs), which is characteristic for this disease, showing several or unique cytoplasmic empty space. ELCs were more frequent in 3D than in 2D culture conditions and contained Weibel-Palade cytoplasmic bodies, which are exclusive structures of endothelial cells.

**Conclusions:**

Both cell lines, IPC-366 and SUM-149, shared ultrastructural characteristics, further supporting canine IMC as a model for the human disease. To the best of our knowledge, this is the first study that demonstrate the morphological differentiation of cultured cancer stem cells from cancer epithelial cell lines into endothelial-like cells, confirming the vasculogenic mimicry phenomenon from an ultrastructural point of view.

## Background

Human inflammatory breast cancer (IBC) and canine inflammatory mammary cancer (IMC) are the most aggressive mammary neoplasms and are associated to poor prognosis in both species [1–5]. The criterium for histological diagnosis for IBC and IMC is the enormous neoplastic embolization of dermal lymphatic vessels which blockade lymphatic drainage originating the distinctive edema [4, 6–9]. The clinical form is characterized by a sudden presentation of erythema, firmness, warmth and pain resembling an inflammatory process and, therefore, this condition can be misdiagnosed with a dermatitis or mastitis, especially if a mammary nodule is absent [1, 2, 4–7]. Numerous epidemiologic, clinical and histopathological characteristics are shared by IBC and IMC, being the latter a good spontaneous animal model for the study of IBC [5, 10, 11].

Characteristically, exacerbated angiogenesis, lymphangiogenesis, lymphangiotropism and vasculogenic mimicry (VM) are found in IBC and IMC [5, 9, 10, 12, 13]. In order to grow and metastasize, tumors require a proper oxygen and nutrients supply. The angiogenic process (sprouting angiogenesis) is relatively complex and it is regulated by numerous pro- and anti-angiogenic factors, standing out the VEGF family and their receptors [14]. VEGF-A is an angiogenic marker that is overexpressed in IBC/IMC and it is present in normal endothelial cells but also in neoplastic cells [10, 12]. According to our previous study, both cell lines overexpress VEGF-A and contributes to the exacerbated angiogenesis [15]. There is an intensive research going on in order to find effective anti-angiogenesis drugs, and more than 300 angiogenesis inhibitors have been identified [16]. Unfortunately, the efficacy of angiogenesis inhibitors in cancer is limited by resistance mechanisms that are poorly understood [17]. Furthermore, multiple studies have used angiogenesis inhibitors as adjuvant therapy and they have failed to provide significant benefits to patients [18].

Angiogenesis is not an exclusive method to nourish tumor tissues. Besides sprouting angiogenesis, that is induced by VEGF-A and is also found in non-neoplastic tissues, two mechanisms of blood supply and metastasis have been discovered in the last years to be exclusive of highly aggressive neoplasms: vasculogenic mimicry (VM) and vascular co-option (VCO) [17, 18]. In VM, cancer stem cells induce tumor neovascularization by their transformation into endothelial-like cells [19]. In VCO cancer cells closely adhere preexisting blood vessels or capillaries to obtain nutrients and oxygen and further develop sprouting angiogenesis. Hypothetically, both VM and VCO would explain the failure of antiangiogenic therapies while VCO would be essential in the metastatic growth [17, 18].

VM is the formation of vascular channels lined by highly malignant neoplastic cells that gain endothelial cells characteristics and are supposed to play an important role in the mechanisms of tumor invasion and metastasis [19–21]. Initially, vessels formed by VM are lined by a mixed of tumor cells and endothelial cells that gradually transform in tumor cells only. These VM newly formed vessels connect with preexisting vessels [19]. Hence, VM is an auspicious target for the developing of new anti-cancer therapy strategies. VM is prognostic characteristic in human oncology having patients with VM a poor clinical outcome [18, 21].

VM is related to the presence of the so-called endothelial-like cells (ELCs) [9]. Endothelial cells store the procoagulant glycoprotein von Willebrand Factor (vWF) in elongate dense granules, known as Weibel-Palade bodies (WPb) which are key for the identification of endothelial cells by electron microscopy [22].

Several human IBC cell lines such as SUM149, have been established in order to study the *in vitro* mechanisms of this special type of breast cancer [23, 24]. Similarly, the IPC-366 is the unique canine IMC cell line established [25] and has demonstrated to be a good model in comparison with its human counterpart SUM149 [15]. Human SUM149 and canine IPC-366 are triple negative (ER-, PR-, HER2-) epithelial cell lines, with high rates of cell growth in adherent (2D) and non-adherent (3D) conditions and metastatic capacity in mice models [15]. The expression of CD146, a marker of endothelial lineage stem cells, has been related in both cell lines to the presence of VM, due to the existence of CD146 positive endothelial-like cells lining the newly-formed VM channels [15]. Nevertheless, according to some authors, these VM cells could not express endothelial cell markers [18, 20].

Mammospheres, clusters of mammary cell lines growing in 3D, are formed by breast cancer stem cells (BCSC) [26] that constitute multipotent cells that have the capacities of self-renewal, differentiation, unlimited growth and can give rise to phenotypically different neoplastic subpopulations [27]. Mammospheres of SUM149 and IPC-366 cell lines exhibit a very similar immunophenotype for the expression of stem cells markers [15]. Microscopic study of 3D cultures and xenotransplanted mice tumors from SUM149 and IPC-366 mammospheres have also revealed the presence of endothelial-like cells (ELCs) indicating that BCSC have the potential to transform into ELCs *in vitro* and *in vivo* (VM) [15]. There is little information regarding ultrastructural characteristics of neoplastic mammary cell lines in adherent conditions (2D) [28–30] and the ultrastructural characteristics of mammospheres (3D) are unknown [31–33]. To the best of our knowledge, there are no previous studies on the ultrastructural features of ELCs neither in cancer tissues nor cancer cell lines.

The aims of this study were to analyze by transmission and scanning electron microscopy (TEM and SEM), the human IBC cell line (SUM149) and the canine IMC cell line (IPC-366) in adherent (2D) and non-adherent (3D) conditions in order to compare the morphological characteristics of both cell lines for the better understanding of their biology and to further support the IPC-366 cell line as a good comparative model for human IBC. Another hypothesis to confirm, is the possible identification of neoplastic epithelial cells showing ultrastructural characteristics of endothelial cells.

## Methods

### Cell lines cultures in adherent conditions

SUM149 triple negative (ER−, PR−, HER-2−) human inflammatory breast carcinoma cell line was obtained from Asterand, Plc. (Detroit, Michigan, USA) in 2015, was maintained in Ham’s F-12 media supplemented with 10% fetal bovine serum (FBS) (Sigma Aldrich, Madrid, Spain),1 μg mL^−1^ hydrocortisone, 5 μg mL^−1^ insulin and 1% penicillin–streptomycin solution and 1% amphotericin B (Sigma Aldrich, Madrid, Spain). Triple negative canine inflammatory mammary carcinoma cell line, established and maintained in our laboratory [25], IPC-366 (commercially available by Applied Biological Materials, ref. T8202) was cultured in Dulbecco’s modified Eagle medium nutrient mixture F-12 Ham (DMEM/F12) containing 10% (FBS), 1% penicillin streptomycin solution and 1% L-glutamine (Sigma Aldrich, Madrid, Spain). Both cell lines were cultured in 25-cm^2^ culture flasks and maintained in a humidified atmosphere of 5% carbon dioxide at 37^∘^C. The cell cultures were observed daily by a phase-contrast microscopy to check cell viability and growth.

### Cell lines cultures in non-adherent conditions: mammosphere formation assay

In order to obtain the primary mammospheres, SUM149 and IPC-366 adherent cells were trypsinized, and the resultant single cells were seeded in 6-well ultra-low attachment plates (1×10^4^ and 2×10^4^ cells mL^−1^)(Corning; New York, NY, USA) [23, 26, 34] in serum-free MEM supplemented with 20 ng mL^−1^ bFGF (basic fibroblast growth factor), 20ng mL^−1^ EGF (epidermal growth factor) and 1× B27 (serum-free supplement) (Invitrogen, Madrid, Spain) enriched media and incubated for 7 days. Then, the mammospheres were stained with MTT [3-(4,5-dimethylthiazolyl-2)-2, 5-diphenyltetrazolium bromide] (Invitrogen, Madrid, Spain) to improve visualization before they were counted using a Gel-count colony counter (Oxford Optronix, Oxford, UK). After 1 week of culture, the first generation of mammospheres were harvested from the cultures and counted with a minimum size of 50 μm. The resulting mammospheres were dissociated into single cells, re-cultured through passages and counted every week.

### Transmission electron microscopy

For the TEM, eight pellets were obtained (two for each cell line and type of culture adherent and non-adherent) and fixed with 2.5% glutaraldehyde (EMS) and 4% paraformaldehyde (EMS) solution. Then, the cells were incubated with 0.1 M Milloning´s buffer 4°C overnight, treated with 2% osmium tetroxide (Panreac) and 3% ferrocyanide (Panreac) solution (diluted in PBS) for 1h. Subsequently, they were washed with distilled water and dehydrated in acetones of increasing percentage (30, 50, 70, 80, and 100%). The samples were gradually infiltrated in a Müllenhauer mixture resin (SPURR resin, TAAB), and solidified at 60 8°C for 48h. The embedded cells were ultrasectioned, observed and photographed at the National Electron Microscopy Center (Madrid) by means of a JEOL JEM 1010 transmission electron microscope.

### Scanning electron microscopy

The mammospheres of each cell line contained in a 6-well ultra-low attachment plate were fixed for 3 hours at 4ºC in 4 % paraformaldehide and 2,5% glutaraldehyde/0,1 M Milloning’s buffer (pH 7.2). Cells were washed twice in distilled water and post-fixed for 1 hour in buffered 1% osmium tetroxide. The samples were dehydrated in an ascendant series of ethanol solution (30%, 50%, 70%, 80% and 100%). Finally, the samples were dried using a critical point dryer (Leika EM CPD 300). The dried samples were sputtered with a 6 nm layer of gold using a Quorum Q150RS. Observation and photographs were made using a JEOL JSM 6400 scanning electron microscope.

## Results

### Transmission Electron Microscopy (TEM)

#### Cell lines cultures (SUM149 and IPC-366) in adherent conditions (2D)

**Fig. 1:**
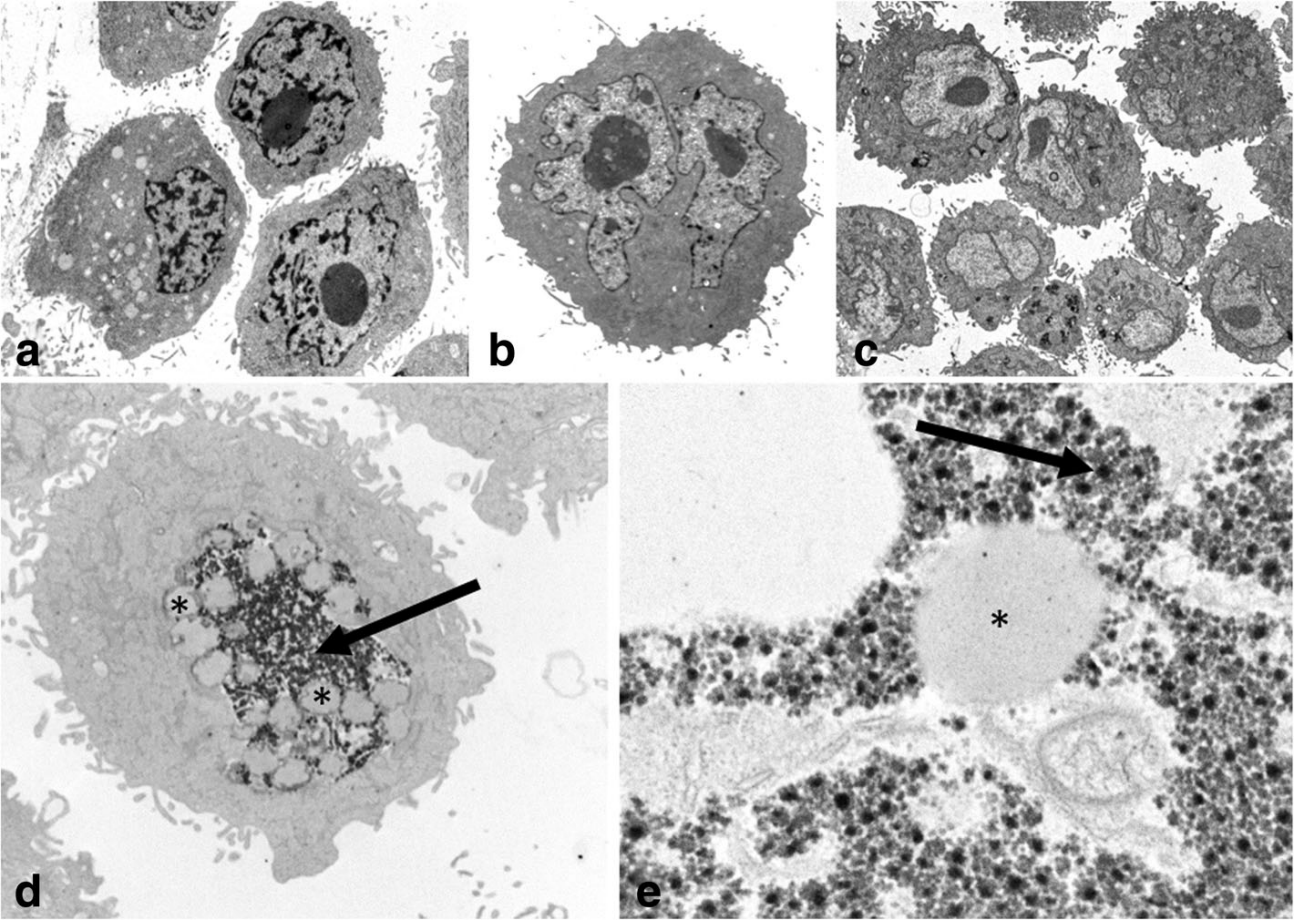
Transmission electron microscopy of SUM149 (**a**, **b**) and IPC-366 (**c**) in adherent conditions (2D). Large individualized round cells showing cytoplasmic membrane processes (microvilli) and marked anisocaryosis and anisocytosis and prominent nucleoli. **d**, **e** IPC-366. Peroxisomes (arrow) in close apposition to lipid droplets (asterisks). Original magnification; **a**, **b** × 6,000, **c** × 4,000, **d** × 12,000, **e** × 50,000

The pellets from the SUM149 and IPC-366 cell lines in adherent cultures, shared very similar characteristics. Both cell lines contained a majority of large individualized cells and some groups of joint round to oval cells, showing several malignant features such as: marked anisocytosis and anisocaryosis, with varying nuclear-cytoplasmic ratios, one or two prominent nucleoli and some atypical mitoses. Binucleate and multinucleated cells were frequently observed. All cells exhibited numerous well developed “digit-like” microvilli or cytoplasmic processes at the cytoplasmic membrane, which did not contain actin or myosin filaments (Fig. 1).

Inside the cytoplasm, high numbers of clear lipid droplets surrounded by numerous spheroid organelles (150-250 nm), containing a slightly electron dense matrix with fine granules (peroxisomes and microperoxisomes) were frequent. In some cases, peroxisomes contained an electron dense core (crystalline catalase/uric acid oxidase) (Fig. 1).

**Fig. 2:**
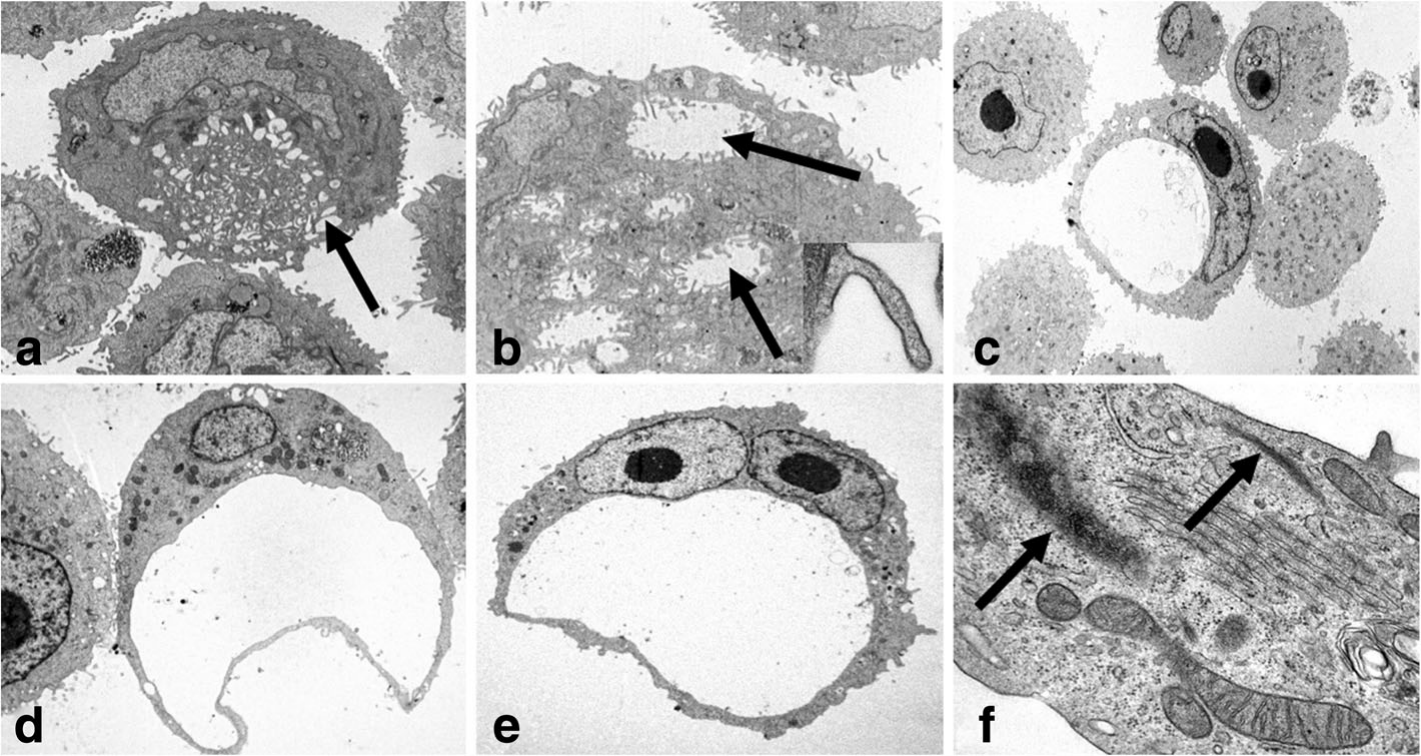
Transmission electron microscopy of IPC-366 (**a**, **b**) in adherent conditions (2D) and SUM149 (**c**, **d**, **e**, **f**) in non-adherent conditions (mammospheres). **a** and **b**: Endothelial-like cells (ELCs) in formation. Multiple empty cytoplasmic spaces (arrows), with microvilli covered by cytoplasmic membrane (insert) and nucleus margination. **c**, **d** and **e**: ELCs showing the characteristic morphology: a unique cytoplasmic empty space and eccentric nucleus. **f**: ELC cytoplasm with Weibel-Palade bodies (arrows). Original magnification; **a**, **d**) × 6,000, **b**) × 10,000, **c**) × 3,000, **e**) × 4,000, **f**) × 60,000

A hallmark finding in both cell lines was the presence of cells with a large unique or small multiple coalescent cytoplasmic clear empty spaces surrounded by cytoplasmic membrane with an elongated eccentric nucleus or nuclei, resembling morphologically a single–endothelial cell capillary vessel (endothelial-like cells, ELCs) (Fig. 2).

#### Cell lines cultures (SUM149 and IPC 366) in non-adherent conditions (3D)

**Fig. 3:**
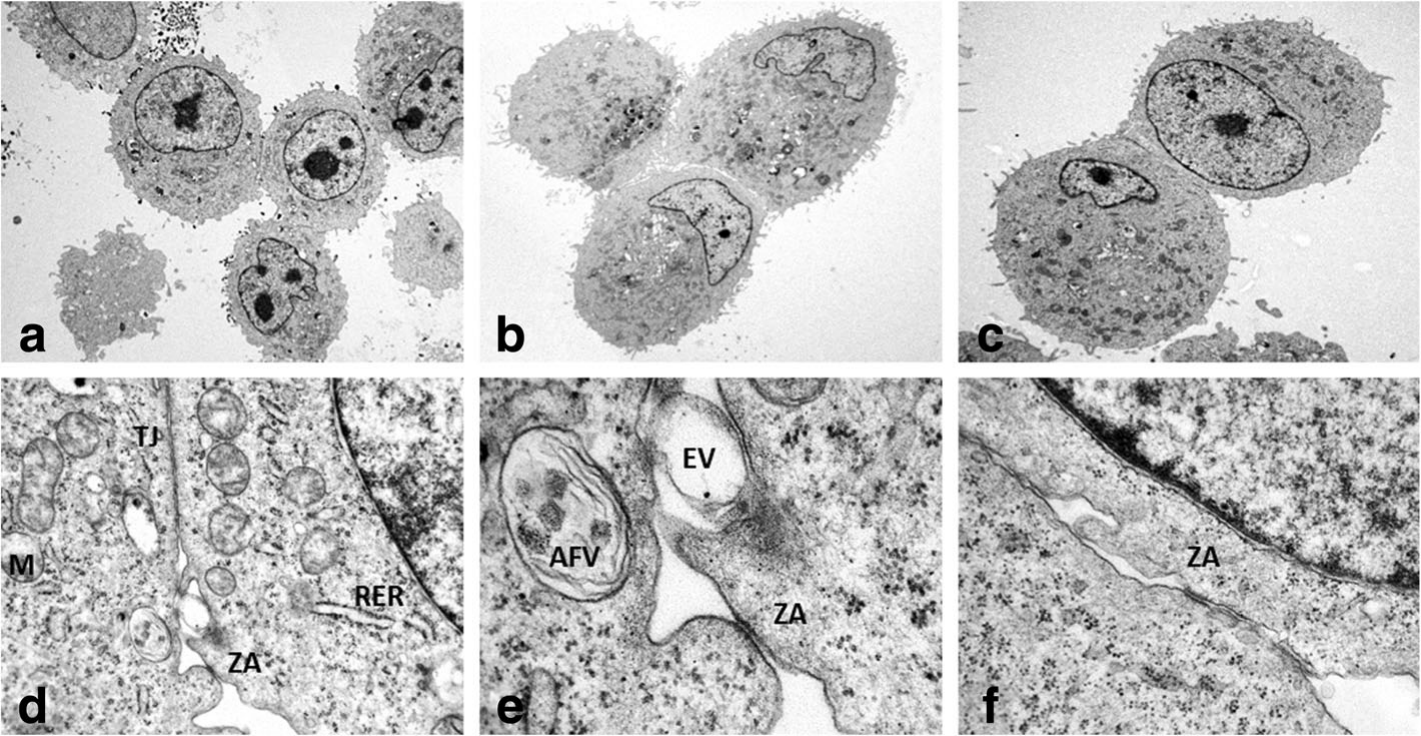
Transmission electron microscopy of IPC-366 (**a**, **d**, **e**) and SUM 149 (**b**, **c**, **f**) mammospheres. **d** is magnified in **e**. Groups of joined cells by tight- junctions (TJ) and belt desmosomes (zonula adherens, ZA). Rough endoplasmic reticulum (RER). Swollen and degenerate mitochondrias (M). Autophagic vacuole (AFV). Membrane-derived vesicle (EV). Original magnification; **a**, **b**) X 4,000, **c**) X 6,000, **d**) X 30,000, **e**) X 100,000, **f**) X 60,000

Ultrastructural features of SUM 149 and IPC-366 in non-adherent cultures (mammospheres) were very similar, but differed from their adherent counterpart in the presence of groups of cells (Fig. 3) and the existence of more abundant endothelial–like cells (ELCs).

Higher magnification of the group of cells revealed the intercellular junctions: D) tight-junctions and E) zonula adherens. In tight junctions, also named zonula occludens, lateral cell cytoplasmic membranes of two adjoining cells come together and fuse with resultant obliteration of the intercellular space. In zonula adherens, also named belt desmosome, the intercellular space (approximately 200 A) is occupied by homogeneous, apparently amorphous material of low density, and there are conspicuous bands of dense material in the subjacent cytoplasmic matrix (Fig. 3). True desmosomes were not observed.

In general, the cytoplasms contained abundant organelles (mitochondria, Golgi apparatus (G), rough endoplasmic reticulum (RER) abnormally distributed and frequently swollen and degenerated. Nuclei were frequently irregular and indented in shape, with predominant euchromatin and less abundant heterochromatin, mostly attached to the inner nuclear membrane. Abundant intermediate filaments, up to 10 nm diameter, were also present. Scattered autophagic vacuoles with double membranes containing remains of cellular organelles and abundant myeloid bodies were present. Some neoplastic cells created and shed external round membrane vesicles, identified as extracellular derived vesicles (EVs), specifically exosomes (up to 50-60 nm in diameter). Exosomes were detected in the cytoplasm, close to the cell membranes or in the extracellular medium encircled by cytoplasmic processes (Fig.3).

Some ELCs in mammospheres contained intracytoplasmic tubular elongated membrane-bound structures, measuring up to 2000-3000 nm in length and 200 nm thick, showing parallel alignment of internal striations identified as Weibel-Palade bodies (WPb) (Fig. 2).

### Scanning Electron Microscope (SEM)

#### Cell lines cultures (SUM149 and IPC-366) mammospheres

**Fig. 4:**
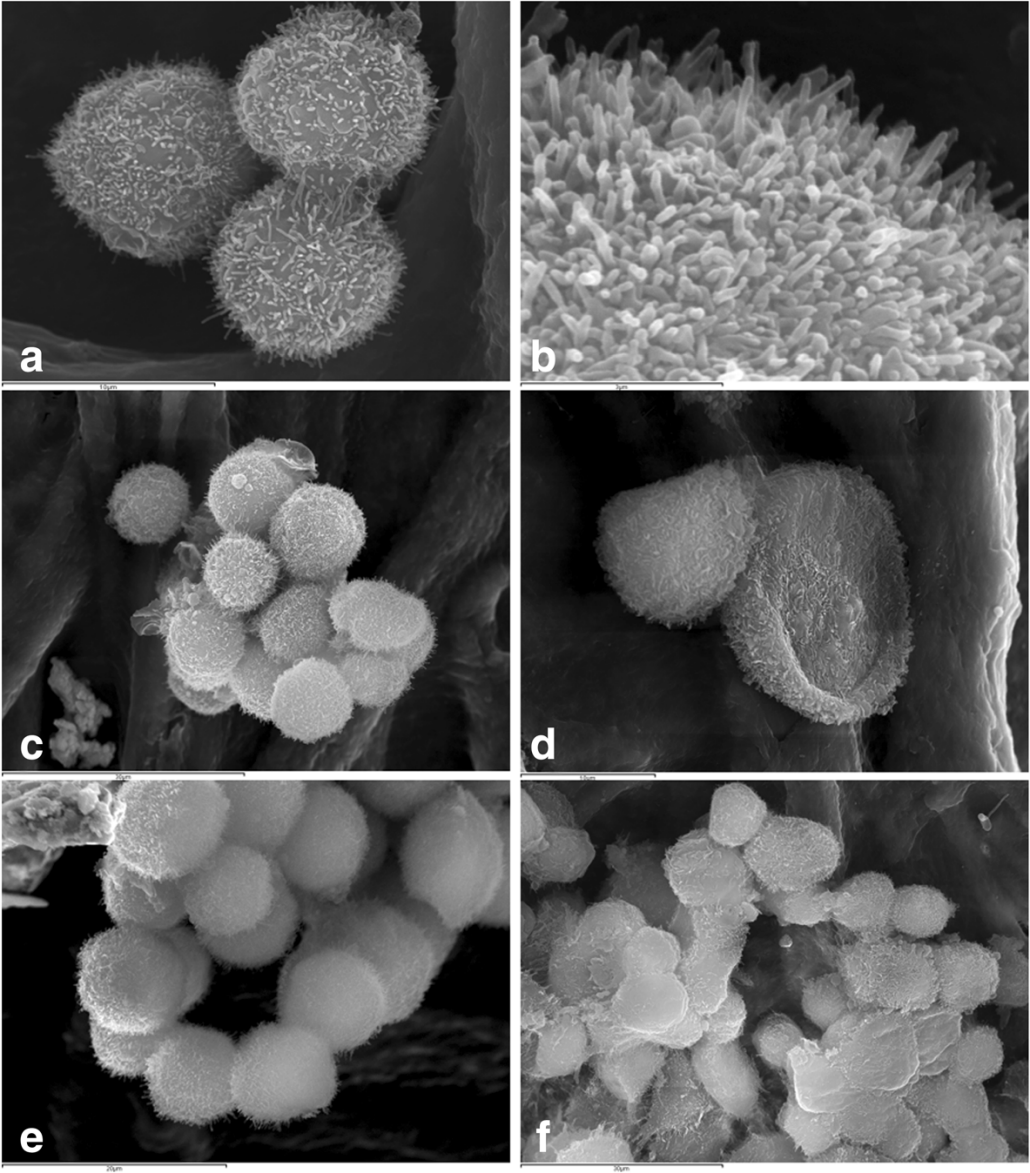
Scanning electron microscopy of IPC-366 (**a**, **b**, **c**, **e**) and SUM149 (**d**, **f**) mammospheres. **a** Joint cells covered by numerous cytoplasmic projections (microvilli). **b** Magnification of microvilli. **c** and **d** Spherical and flattened cells, respectively. **e** and **f** Mammary acini-like structures

Mammospheres of both cell lines showed groups of cells with multiple cytoplasmic projections over the surface. Occasionally, these structures appeared arranged around a lumen-like structure and less frequently the cells appeared isolated. There were two cellular shapes: rounded and flattened cells. The surface of some cells seemed to have extruded through the membrane boundary, originating plasma membrane blebs (Fig. 4).

## Discussion

Human inflammatory breast cancer (IBC) and canine inflammatory mammary cancer (IMC) are comparable diseases [5, 10, 11]. IBC/IMC is a very aggressive type of breast cancer with poor prognosis [1–5]. IBC/IMC has specific carcinogenic mechanisms, including high rates of metastasis and invasiveness that still are poorly understood. In order to study the “inflammatory” phenotype from a mechanistic point of view, several IBC (i.e. SUM 149) cell lines have been established [23, 24]. IPC-366, a canine IMC cell line, has been demonstrated to share similar characteristics with its human counterpart, the IBC cell line SUM149 [15]. The literature regarding ultrastructural features of mammary cell lines is scant [28–30, 32]. To the best of our knowledge, this is the first report in which human and canine inflammatory mammary cell lines are ultrastructurally compared in adherent (2D) and non-adherent (3D) conditions. Few studies refer the ultrastructural morphology of the IBC mammospheres [31, 33].

In highly malignant neoplasms, the presence of vascular channels lined up by disregulated neoplastic cells has been found and defined as vasculogenic mimicry (VM) [35]. VM was firstly described in human melanoma [20] and has been found to be frequent in IBC/IMC. VM has been identified in both cell lines (SUM149, IPC-366), showing cells with endothelial-like morphology (ELCs) [15]. SUM-149 and IPC-366 cells have the potential to differentiate into endothelial-like cells (ELCs) *in vitro* and *in vivo* [15]. The ability of cancer stem cells to transform into endothelial cells has been previously reported [36]. In the present study, both cell lines, 2D and 3D, contained cells with a large unique cytoplasmic empty space that marginated the nuclei to the periphery resembling one capillary endothelial cells (endothelial-like cells, ELCs) [9]. Other cells had several small cytoplasmic empty spaces, interpreted as forming ELCs, according to the previously published ultrastructural morphology of endothelial cells in formation [37], although their morphology has not been studied yet. By SEM, two cellular shapes appeared: rounded and flattened cells. The latter ones are compatible with endothelial-like cells.

The present descriptive study can only address the morphology of the cells, however, there are previous studies on these two cell lines that support the molecular transformation of these cultured cells, with stem cells phenotype, into ELCs [15, 25]. IPC-366 cells, including ELCs, were intensely positive for COX-2 [25], which is considered a marker for ELCs involved in VM [10, 38] and a stem cell marker [39]. Moreover, SUM149 and IPC-366 expressed CD146 [15], a cell adhesion molecule specific marker for endothelial cell lineage [40]. Nevertheless, according to previous studies, it is possible that the VM cells would not be able to express endothelial cell markers [18, 20]. ELCs immunostaining with CD31 in IMC primary tumors, was inconclusive, and considered mostly negative [9]. The negative result of the ELCs for CD31 is in agreement with previous similar studies in human intraocular melanoma [20] and human IBC xenograft [41] . Furthermore, in several human clinical studies, the presence of CD31+ cells in VM is controversial [18].

According to the present results, both cell lines can acquire also unequivocal ultrastructural features of endothelial cells, since some ELCs in mammospheres exhibited Weibel-Palade cytoplasmic bodies (WPb). By definition, WPb are specific endothelial cells cytoplasmic structures that store von Willebrand factor (vWF) that is required for correct hemostasis [42, 43]. WPb has also a role in inflammation, vascular distention and angiogenesis [44]. Furthermore, vWF and WPb formation are regulated by the RER and G complex [44]. Accordingly, WPb often appeared in close apposition to RER and G complex.

Excluding the ELCs, the rest of neoplastic cells of both cell lines had similar morphological features as previously published in non IBC/IMC breast cancer cell lines by means of transmission and scanning electron microscopy [28–33, 45].

The results of the present study revealed that both cell lines have similar ultrastructural features; by transmission electron microscopy (TEM), in 2D and 3D cultures. Both, SUM149 and IPC-366 cell lines were round to oval cells with numerous surface microvilli, a high nuclear-cytoplasmic ratio, marked anisocytosis and anisocaryosis, abundant peroxisomes and the presence of frequent highly malignant multinucleated cells and endothelial-like cells (ELCs). Although normal mammary epithelial cells have cytoplasmic microvilli, it has been exhibited by TEM and Scanning Electron Microscopy (SEM) that both cell lines presented an exacerbated formation of microvilli over the surface. This special feature represents a dramatic increase of the cell surface and could be a reflection of a more malignant, efficient or abundant connection from the cells to the external medium [46, 47] . The characteristic presence of euchromatin is predominant in cancer cells and is attributable to the high percentage of cells in DNA synthesis phase (S phase) [48].

An interesting finding observed in both cell lines was the intracytoplasmic high number of peroxisomes closely located to lipid droplets. Peroxisomes have an important role in the lipid metabolism. These organelles contain large amounts of oxidases that catalyze the oxidation of long chain saturated fatty acids to acetyl- CoA [49, 50]. In general, great amount of peroxisomes are found in cells that synthetize, metabolize or store lipids and/or steroid hormones, such as cells of the adrenal gland cortex, Leydig-cells, corpus-luteum-cells, fat cells and epithelial cells of the gut [51]. A significant high content of steroid hormones have been indicated in tumor samples and serum of dogs with IMC [52–54]. Also, the secretion of steroid hormones (progesterone, estrone sulfate, estradiol, androstenedione and testosterone) by SUM149 and IPC-366 in vitro cell lines has been recently described [55]. Thus, also could explain the high content of cytoplasmic peroxisomes in SUM149 and IPC-366.

By TEM it was observed that cells of SUM149 and IPC-366 mammospheres were frequently joined together by tight junctions and belt desmosomes (zonula adherens). The cell to cell epithelial molecule adhesion E-cadherin is typically present in zonula adherens associated with intracellular actin microfilaments [56]. Interestingly, in contrast with other metastatic epithelial cancers that loss E-cadherin, IBC typically overexpress E-cadherin in the metastatic process [57, 58]. IBC cell line SUM149 [59] and IMC cell line IPC-366 [25] also overexpress E-cadherin. By SEM, both cell lines mammospheres showed groups of joined cells, and frequently appeared as acini-like structures with a central lumen.

Extracellular derived vesicles (EVs) are membrane-limited vesicles that are released into the extracellular microenvironment that are abnormally increased in cancer cells [60, 61]. Their role is still unknown; EVs contain diverse small molecules as proteins, lipids, microRNAs, mRNA and DNA fragments [62] and participate in intercellular communication [63]. The knowledge about the EVs is rapidly expanding and they are considered important as potential breast cancer biomarkers and therapeutic targets [64]. In cancer, EVs promote proliferation [65–67], migration [68], angiogenesis [69], invasion and metastases [68], as well as induction of epithelial-to-mesenchymal transition (EMT) [70]. In the present study, abundant number of EVs in SUM149 and IPC-366 mammospheres were detected by TEM. Additionally, by SEM, small round vesicles extruded on the surface were observed; this structures are considered compatible with EVs according to the size of the vesicles (from 50 nm to 2 μm) and some of them were identified as apoptotic bodies [71]. Stem cells are an abundant source of EVs [61]. As previously reported, SUM149 and IPC-366 cell lines in non-adherent (3D) cultures, exhibited similar immunophenotype for the expression of stem cells markers. In veterinary medicine, very little is known on cancer‐derived EVs. There is only a preliminary investigation on extracellular vesicles in canine and feline mammary cancer [72]. Further studies are necessary to isolate, identify and characterize EVs from IBC/IMC cell lines.

## Conclusions

In summary, this investigation has provided evidence that SUM-149 and IPC-366 share ultrastructural characteristics, supporting canine IMC as a model for the human disease. This study revealed for the first time, the morphological differentiation of cultured cancer stem cells from epithelial cell lines into endothelial- like cells, showing ultrastructural characteristics of endothelial cells and confirming the presence of the vasculogenic mimicry phenomenon.

## Data Availability

All samples and photographs are stored at the National Electron Microscopy Center and the Dept. of Animal Medicine and Surgery, Veterinary School, University Complutense of Madrid.

## Abbreviations

2D: Adherent conditions
3D: Non-adherent conditions
AFV: Autophagic vacuole
BCSC: Breast cancer stem cell
bFGF: Basic fibroblast growth factor
DMEM: Dulbeccos’s modified Eagle medium
EGF: Epidermal growth factor
ELCs: Endothelial-like cells
EMT: Epithelial-to-mesenchymal transition
ER: Estrogen receptor
EVs: Membrane-derived vesicles
FBS: Fetal bovine serum
G: Golgi apparatus
HER2: Human epidermal growth factor receptor
IBC: Inflammatory breast cancer
IMC: Inflammatory mammary cancer
M: Mitochondria
MEM: Minimum Essential Medium
PBS: Phosphate-buffered saline
PR: Progesterone receptor
RER: Rough endoplasmic reticulum
SEM: Scanning electron microscopy
TEM: Transmission electron microscopy
TJ: Tight junction
VCO: Vascular co-option
VM: Vasculogenic mimicry
VWF: Von Willebrand Factor
WPb: Weibel- Palade body
ZA: Zonula adherens
